# Yttria-Stabilized Zirconia Composite Coating as Barrier to Reduce Hydrogen Permeation into Steel

**DOI:** 10.3390/ma17123017

**Published:** 2024-06-20

**Authors:** Jianmeng Wu, Jiaqi Xie, Mengyuan He, Jingyi Zhang, Songjie Li

**Affiliations:** School of Chemical Engineering, Zhengzhou University, Zhengzhou 450001, China; 17803875727@163.com (J.W.); xiejiaqi202103@163.com (J.X.); 18737135837@163.com (M.H.); jingyzhang2002@163.com (J.Z.)

**Keywords:** hydrogen, coating, crystalline stabilization, zirconia, permeation

## Abstract

Hydrogen atoms can enter into metallic materials through penetration and diffusion, leading to the degradation of the mechanical properties of the materials, and the application of hydrogen barrier coatings is an effective means to alleviate this problem. Zirconia coatings (ZrO_2_) have been widely studied as a common hydrogen barrier coating, but zirconia undergoes a crystalline transition with temperature change, which can lead to volumetric changes in the coating and thus cause problems such as cracking and peeling of the coating. In this work, ZrO_2_ coating was prepared on a Q235 matrix using a sol-gel method, while yttria-stabilized zirconia (YSZ) coatings with different contents of rare earth elements were prepared in order to alleviate a series of problems caused by the crystal form transformation of ZrO_2_. The coating performances were evaluated by the electrochemical hydrogen penetration test, pencil hardness test, scratch test, and high-temperature oxidation test. The results show that yttrium can improve the stability of the high-temperature phase of ZrO_2_, alleviating the cracking problem of the coating due to the volume change triggered by the crystalline transition; improve the consistency of the coating; and refine the grain size of the oxide. The performance of YSZ coating was strongly influenced by the yttria doping mass, and the coating with 10 wt% yttria doping had the best hydrogen barrier performance, the best antioxidant performance, and the largest adhesion. Compared with the matrix, the steady-state hydrogen current density of the YSZ coating decreased by 72.3%, the antioxidant performance was improved by 65.8%, and the ZrO_2_ coating hardness and adhesion levels were B and 4B, respectively, while YSZ coating hardness and adhesion were upgraded to 2H and 5B. With the further increase in yttrium doping mass, the hardness of the coating continued to improve, but the defects of the coating increased, resulting in a decrease in the hydrogen barrier performance, antioxidant performance, and adhesion. In this work, the various performances of ZrO_2_ coating were significantly improved by doping with the rare earth element, which provides a reference for further development and application of oxide coatings.

## 1. Introduction

As the most widely distributed substance in nature, hydrogen energy is regarded as the clean energy with the most development potential in the 21st century for its high calorific value, lack of pollution, and abundant sources [1,2,3]. Hydrogen energy is conducive to promoting the clean and efficient use of traditional fossil energy and supporting the large-scale development of renewable energy [4,5]. However, for metal materials in a hydrogen environment, due to the small radius and high permeability of hydrogen atoms, their entry into the metal material leads to a decline in the mechanical properties of the material. The plasticity of the material especially is reduced, triggering the occurrence of hydrogen damage or even fracture phenomena, resulting in huge economic losses or even a disaster [6,7,8].

American scholar Flowler first proposed the concept of hydrogen permeation barrier coatings at the end of the 1970s [9]. Many studies have shown that hydrogen barrier coatings on the surface of metal materials can effectively slow down the diffusion of hydrogen into the metal materials, which can reduce the occurrence of hydrogen damage phenomena and improve the safety of the whole process of hydrogen energy utilization [10,11,12]. According to the type, the hydrogen barrier coating can be divided into oxide coatings [13,14], carbide coatings [15,16], and metal alloy coatings [17]. Among them, the oxide coating has the advantages of stable chemical properties, good wear resistance, low solubility and permeability of hydrogen, low cost, etc.; oxide coatings are the most widely studied type of coating, including chromium oxide coating [18,19], alumina coating [20], and so on. For example, He et al. [21] prepared a Cr_2_O_3_ coating on 316L stainless steel by the metal–organic decomposition (MOD) method, using chromium acetylacetonate as the precursor. The hydrogen permeation results show that the hydrogen permeability of Cr_2_O_3_ coating can be reduced by 24–117 times at 823–973 K. Feng et al. [22] deposited Al_2_O_3_ as a hydrogen barrier coating on a 316L matrix by plasma electrolytic oxidation, and the results of gas-phase hydrogen permeation tests showed that the alumina coating was able to reduce the rate of hydrogen permeation by three orders of magnitude concerning the pure matrix. However, the thermal expansion coefficient between the metal matrix and the oxide coating differs greatly, and there is an obvious thermal mismatch problem, which is highly likely to lead to problems such as cracks and coating peeling. Zhang et al. [23] prepared an Al_2_O_3_ coating on a 316L matrix by the MOD method, but due to the difference in thermal expansion coefficients, cracks and defects appeared in the Al_2_O_3_ coating during the drying and annealing process, and the protective effect of the coating on the matrix was improved by sealing and densifying the coating with aluminum phosphate.

Certainly, the selection of an oxide material with a coefficient of thermal expansion similar to that of the matrix is an important means of improving the hydrogen barrier properties of the coating. In recent years, zirconia (ZrO_2_) has been widely studied as a new type of hydrogen permeation barrier coating material with a coefficient of thermal expansion much closer to those of various types of steel materials [24,25], and the diffusion coefficient of hydrogen in zirconia is low [26,27,28]. Hatano et al. [29] prepared ZrO_2_ coatings on ferrite steel by the sol-gel method and electrolytic deposition technique, and the coatings showed a good hydrogen barrier effect at 300–550 °C according to the gas phase hydrogen permeation test. However, ZrO_2_ is a polycrystalline oxide, and as the temperature changes, the crystalline form will shift, which will lead to changes in the volume of ZrO_2_ and thus cause cracking and peeling of the ZrO_2_ coating [30]. Therefore, adopting reasonable preparation methods and processes to inhibit the transformation of the ZrO_2_ crystal type and reduce the cracking phenomenon of the coating is an important means to promote the wide application of ZrO_2_ in the field of hydrogen permeation barrier coating [31,32]. The radius difference between yttrium (Y) ions and zirconium ions is smaller than that of other metals, so yttrium has been used to stabilize zirconium oxide crystals in many studies, and excellent results have been achieved [33].

Based on the above viewpoints, in this work, ZrO_2_ coatings were prepared on a Q235 mild steel matrix by the sol-gel method. Meanwhile, in order to alleviate the problems of coating cracking and peeling caused by the crystalline transformation of ZrO_2_, as well as to improve the hydrogen barrier performance and other comprehensive properties of the coatings, yttrium nitrate with different contents was introduced into the coating preparation process. The effects of the addition of the Y element on the surface morphology, hardness, bonding force, antioxidant performance, and hydrogen barrier performance of the coatings were investigated.

## 2. Experimental Procedures

### 2.1. Materials

n-propanol (>99.5 wt%), n-propanol zirconium (70 wt% in n-propanol), acetylacetone (>99.0 wt%), and yttrium nitrate (>99.5 wt%) were supplied by Shanghai Macklin Biochemical Technology Co., Ltd. (Shanghai, China). Sodium sulfide (>99.0 wt%) and sodium hydroxide (>95.0 wt%) were purchased from Shanghai Aladdin Reagent Co., Ltd. Acetone (>99.0 wt%) and anhydrous ethanol (>99.5 wt%) were obtained from Sinopharm Chemical Reagent Co., Ltd. (Shanghai, China). The dimension of Q235 steel used in this work was 35 mm × 45 mm × 0.3 mm, and its specific composition is shown in Table 1.

### 2.2. Pretreatment of Matrix Q235 Mild Steel

The Q235 matrix was sequentially polished using 1200, 2400, and 4000 mesh sandpaper, followed by polishing of the matrix using a 5 μm diamond polish. Prior to the preparation of the coating, the matrix needed to be ultrasonically cleaned in anhydrous ethanol and acetone sequentially for 20 min and dried to remove oils and impurities from the matrix surface [34,35].

### 2.3. Preparation of ZrO_2_ Coating and Yttria-Stabilized Zirconia Coating

Precursor sols of ZrO_2_ were prepared using n-propanol zirconium as the zirconium source, n-propanol as the solvent, acetylacetone as the complexing agent, and yttrium nitrate as the stabilizer, with a molar ratio of n-propanol zirconium, n-propanol, acetylacetone, and deionized water of 1:10:2:2.5. The contents of yttrium nitrate added (calculated as the mass ratio of Y_2_O_3_ relative to ZrO_2_) were 5 wt%, 10 wt%, and 15 wt%. Firstly, n-propanol zirconium was dissolved in the n-propanol solvent and stirred well. After that, acetylacetone and deionized water were added sequentially and mixed well. After stirring and reacting for 24 h, the resulting sol was placed in a beaker, and the precursor sol of yttria-stabilized zirconia (YSZ) was obtained by sealing and static aging for 48 h at room temperature.

The prepared sol was uniformly coated onto the treated matrix, which was subsequently placed in a drying oven at 85 °C for 20 min to evaporate excess water and solvent in the wet gel. At the end of drying, the specimen was taken out to be coated with the sol again, and the process was repeated three times. The dried specimen was placed in a muffle furnace and heated up to 300 °C at a heating rate of 1 °C/min, and it was taken out after pre-sintering for 35 min; then, the coating and drying process was carried out again, which was repeated three times. Finally, the specimen was placed in a muffle furnace and heated up to 550 °C for heat treatment; the specific process of heat treatment is shown in Table 2. The ZrO_2_ and YSZ coatings were obtained after heat treatment and natural cooling to room temperature in the furnace. The YSZ coatings were named 5YSZ, 10YSZ, and 15YSZ based on the order of Y doping mass from 5 wt% to 15 wt%.

### 2.4. Characterization of Materials

The TG-DTG-DSC curves of the gels were measured using a Differential Thermo-Thermogravimetric Simultaneous Analyzer (Netzsch STA449F5, Bavaria, Germany). With N_2_ as the protective gas, the test temperature range was from room temperature to 800 °C, and the heating rate was 10 °C/min. The physical phase composition of the coatings was analyzed using a X-ray diffractometer (PANalytical Empyrean, Almelo, the Netherlands), which uses a Cu target with a test angle range of 20–80° and a scanning speed of 1°/min. The scanning electron microscope (ZEISS sigma 500, Oberkochen, Germany) was used to observe the microscopic morphology and cross-sectional morphology of the coatings, and a energy dispersive spectrometer (BRUKER XFlash 6130, Billerica, MA, USA) was used to perform surface scanning of the coating surfaces to analyze the composition and ingredients of the film layers.

### 2.5. Hydrogen Barrier Performance Testing of Coatings

**Figure 1:**
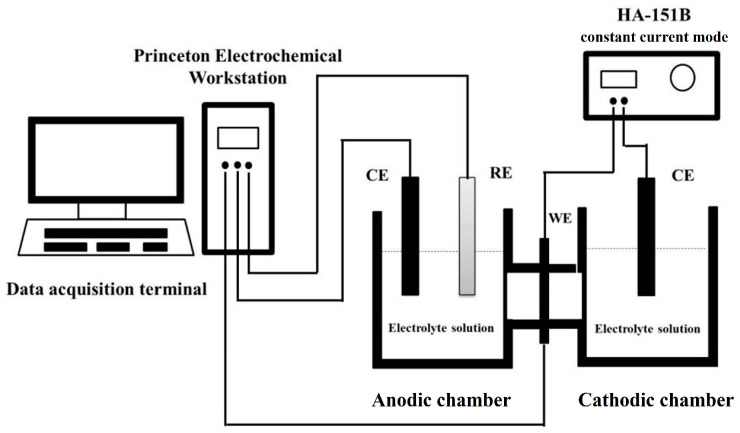
Schematic diagram of electrochemical hydrogen penetration test device.

In this work, electrochemical hydrogen permeation was used to evaluate the hydrogen barrier performance of the coatings. The schematic diagram of the electrochemical hydrogen permeation test setup is shown in Figure 1. The main experimental setup consisted of a Devanathan–Stachurski double electrolytic cell, which contained a cathode chamber and an anode chamber, with the sample to be tested sandwiched between the two chambers. The electrolyte in the cathode chamber was a mixed solution of 0.2 mol/L NaOH and 1 g/L Na_2_S, and the electrolyte in the anode chamber was a 0.2 mol/L NaOH solution. The platinum sheet electrode acted as the auxiliary electrode in the cathode and anode chambers, the Hg/HgO electrode was used as the reference electrode in the anode chamber, and the specimen acted as a double working electrode, both as an anode and cathode. During the experiment, the side with the coating faced the cathode chamber, which was connected to the constant current apparatus, and the anode chamber was connected to the electrochemical workstation. Five parallel tests were performed for each type of coating, and coating specimens whose experimental results varied within 5% were selected as the raw data for electrochemical hydrogen permeation curve plotting.

### 2.6. Hardness and Adhesion Testing of Coatings

The hardness of a coating is one of the most important properties indicating the mechanical strength of the coating and is also an important indicator of the quality of the coating [36]. The coating hardness test was conducted with the Sichuan Strubic BK636 pencil hardness tester (Chengdu, China); the hardness level from low to high was divided into 6B-B, HB, and H-6H, for a total of 13 levels. A vertical pressure of 1 kg was applied to the surface of the coating at an oblique angle of 45° for a length of 3 cm and repeated 3 times. The hardness of the pencil corresponds to the hardness of the coating when the pencil fails to make a scratch.

The strength of the bond between the coating and the matrix largely determines the service life of the coating. The adhesion test was carried out by the Sichuan Strubic BK218 scribe(Chengdu, China), and the adhesion was categorized into six grades from 0B to 5B. The test was conducted with a 218/4 cutter head and a 10 × 10 mm grid of 100 squares. The results were observed using 3 M adhesive tape and a magnifying glass.

### 2.7. Antioxidant Performance Test of Coatings

In addition to the effects of hydrogen atoms on the material, the material is also susceptible to attack by ambient oxygen. In this study, the oxidation resistance of the coated specimens was evaluated by measuring the weight change in the samples before and after they were heated to 500 °C in air and held for different times.

## 3. Results and Discussion

### 3.1. Thermal Transition Temperature Analysis of Different Types of Gel

**Figure 2:**
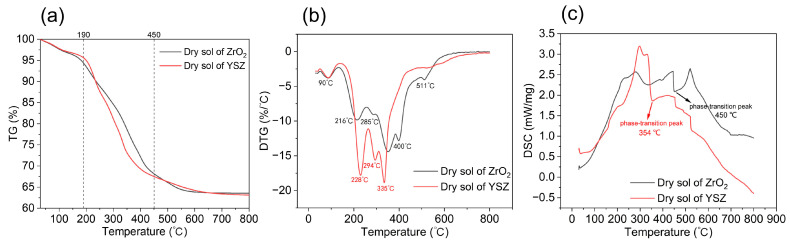
TG (**a**), DTG (**b**), and DSC (**c**) curves of ZrO_2_ and YSZ sol.

The results of the TG-DTG analysis of the dried ZrO_2_ gel powder and YSZ gel powder are shown in Figure 2. From the TG curves in Figure 2a, it can be found that the overall mass loss of the two gels was about 36%, and the mass loss process was divided into three stages. The ZrO_2_ gel lost weight slowly in the range from room temperature to 190 °C, with a mass loss of about 5.7%. In the range of 190–450 °C, the gel lost weight significantly, with a mass loss of 26.2%, and the rate of weight loss was fast. In the range of 450–800 °C, the change in gel mass was small, only 4.6%. YSZ sol had a faster rate of weight loss than ZrO_2_ sol between 190 °C and 450 °C, mainly due to the decomposition of yttrium nitrate.

**Figure 3:**
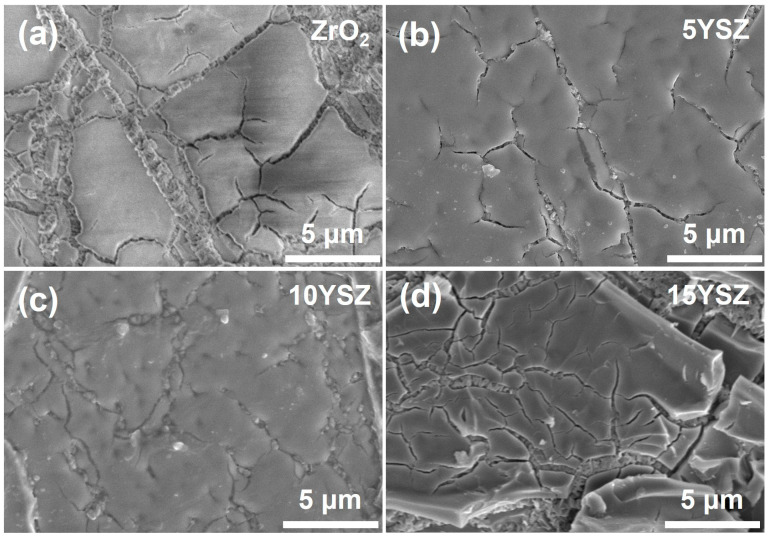
SEM images of (**a**) ZrO_2_ coating, (**b**) 5YSZ, (**c**) 10YSZ and (**d**) 15YSZ coatings surface.

Combined with the DTG curves in Figure 2b, it can be found that both gels showed obvious weight loss peaks near 90 °C, which were mainly attributed to the evaporation of the residual free water and solvent in the gels. Between 190 and −400 °C, the DTG curves showed multiple weight loss peaks. The weight loss peak near 220 °C was mainly attributed to the removal of bound water, and the weight loss peak appeared near 290 °C when the decomposition of organic matter mainly occurred. The weight loss peaks of the DTG curve of ZrO_2_ sol appeared at 400 °C and 511 °C, which was mainly due to the transformation of the ZrO_2_ gel, and the oxides were gradually formed at this stage [37,38]. From the DTG curve of YSZ sol, oxide was formed at a lower temperature and at an accelerated rate, and there was no obvious weight loss peak after 400 °C, which also indicates that Y doping has a positive effect on stabilizing the crystal form of oxides, and the crystalline form of oxides remains stable after formation. From the DSC curves in Figure 3c, it can be seen that the phase transition temperature from the amorphous to the crystalline state of ZrO_2_ gel decreased after the addition of Y element, which indicates that Y can promote the phase transition of ZrO_2_ and stabilize the crystalline form of the oxide to some extent.

### 3.2. Surface Morphology Analysis of Coatings

**Figure 4:**
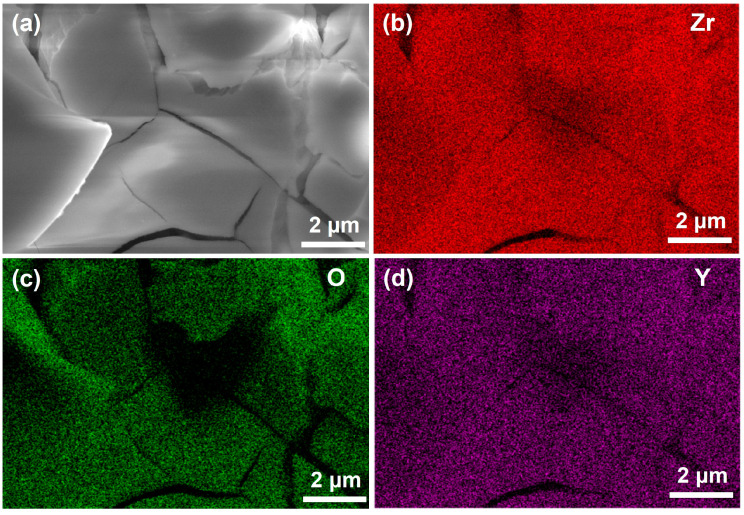
Element distribution on the surface of YSZ coating: (**a**) morphology, (**b**) Zr element distribution, (**c**) O element distribution, (**d**) Y element distribution.

Figure 3 shows the SEM morphology of the ZrO_2_ coating and the YSZ coating, and Figure 4 shows the elemental distribution on the surface of the YSZ coating. From Figure 3a, it can be seen that there are more defects on the surface of the ZrO_2_ coating. The smooth lumpy ZrO_2_ ceramics are uniformly distributed on the surface of the matrix, and a small amount of needle-like iron oxide exists on the surface due to the partial oxidation of the coating during the preparation process [39]. With the doping of Y into the coating, the volume change effect due to the crystallographic transition is suppressed and the cracking of the coating is reduced, but when the doping mass of Y is further increased to 15 wt%, the stability of the crystallographic structure of the oxides deteriorates again. The 10YSZ coating has the densest surface condition with the least number of cracks and defects on the surface.

As can be seen in Figure 4, the YSZ coating is mainly composed of Zr, Y, and O elements; the elements are uniformly distributed in the coating. This indicates that the coating is mainly composed of oxides of Zr and Y, which further indicates the successful doping of element Y into the coating.

### 3.3. Phase Composition Analysis of Coatings

**Figure 5:**
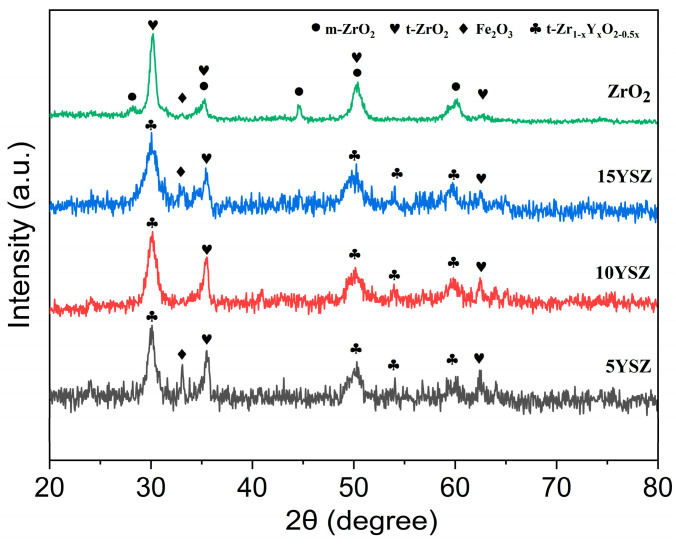
XRD patterns of ZrO_2_ and YSZ coating.

Figure 5 shows the XRD patterns of the ZrO_2_ coating and YSZ coatings. It can be seen that the ZrO_2_ coating is mainly composed of a large amount of monoclinic phase (m-ZrO_2_) and a small amount of tetragonal phase (t-ZrO_2_), along with the Y doping. The monoclinic phase in the ZrO_2_ coatings gradually disappeared, and the coatings mainly contained Y-doped t-ZrO_2_(t-Zr_1−x_Y_x_O_2−0.5x_, 0 < x < 1) and a small amount of t-ZrO_2_ [40], which indirectly proves the successful doping of the Y element.

Meanwhile, at 2θ = 30°, 35°, and 50°, the diffraction peak patterns show a diffuse broadening phenomenon, and the peak intensity decreases, which implies that the YSZ material has a smaller grain size [41,42]. As a whole, the YSZ coating is mainly dominated by tetragonal phase oxides, and the Y doping amount does not have much effect on the physical phase type of the coating, but with the gradual increase in the Y doping content, more and more Y atoms form a composite oxide with Zr atoms, and the content of t-ZrO_2_ decreases gradually while the content of t-Zr_1−x_Y_x_O_2−0.5x_ rises gradually. Diffraction peaks of iron oxide appeared at Y doping levels of 5 wt% and 15 wt%, indicating that there were some defects in the coating during the preparation process under these two doping levels, resulting in oxidation of the matrix when exposed to air. The peak widths of the main diffraction peaks showed a significant reduction in the oxide grain size. It can be calculated from the Scherrer formula that as the Y doping increased from 5 wt% to 15 wt%, the average grain size of the oxides decreased by about 31%, 37%, and 60%, respectively.

### 3.4. Evaluation of Physical Properties of Coatings

**Figure 6:**
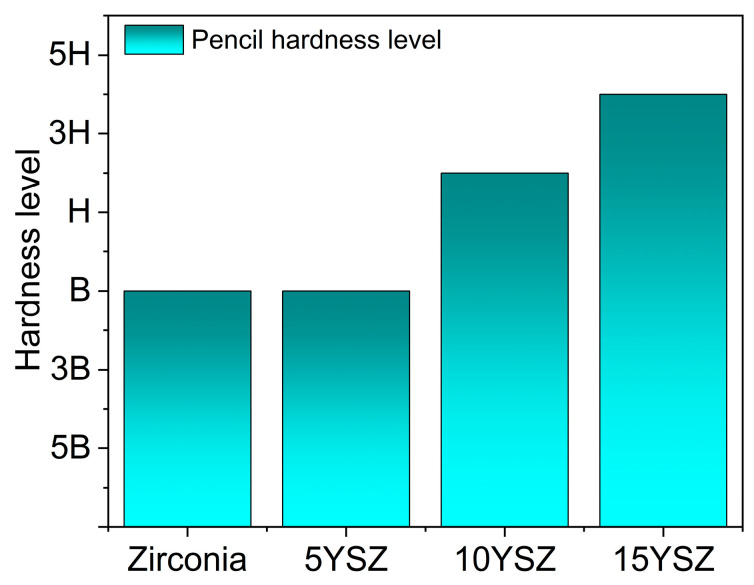
Pencil hardness statistics of ZrO_2_ coating and YSZ coating.

Figure 6 shows the results of the pencil hardness tester for ZrO_2_, 5YSZ, 10YSZ, and 15YSZ coatings. The results show that the hardness level of the ZrO_2_ coatings is B. The hardness of the coatings gradually increases with the increase in Y doping mass, and the hardness level increases from B to 4H. The reason is that with the increase in Y doping, the increase in viscosity and density of the sol makes the thickness of the coating increase under the same coating process. On the one hand, the oxide particles are of high hardness, so the hardness of the coating is enhanced with the increase in the thickness [43]; on the other hand, due to the Y doping making the grain size of the oxide smaller, the effect of grain refinement can effectively improve the hardness and wear resistance of the coating, so the YSZ coating has a greater hardness and enhanced wear resistance compared with the ZrO_2_ coating.

**Figure 7:**
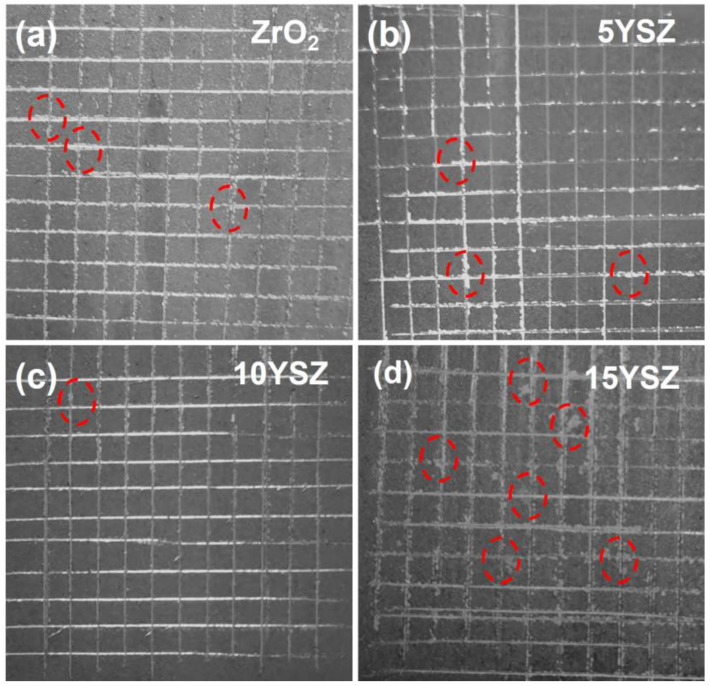
Topography of the (**a**) ZrO_2_ coating, (**b**) 5YSZ, (**c**) 10YSZ and (**d**) 15YSZ coating after scratch test. (Note: The dashed circles in the figure represent the locations where the coating was peeling off).

The results of the adhesion test of the ZrO_2_ coating and the YSZ coating are shown in Figure 7, and the bonding strength of the coatings can be visualized by observing the shedding of the coatings in the scratch test. The scratches were carefully observed with an optical microscope, and it was found that the ZrO_2_ coating had a small amount of separation between the coating and the matrix at the intersection of the scratches and the peeling phenomenon. The overall affected area was less than 5%, and the adhesion test grade was 4B. The 5YSZ coating showed a small amount of the separation and peeling phenomenon between the coating and the matrix at the intersection of the scratches; the overall affected area was less than 5%, and the adhesion test grade was 4B. 10YSZ coating had almost no peeling phenomena at the edge of the scratch; the adhesion test grade was 5B. 15YSZ coating showed a partial peeling phenomenon at the edge of the scratch; the overall impact area was more than 5% but less than 15%, and the test grade was 3B.

The results of adhesion and hardness tests of the coatings show that the addition of Y at a low doping amount can inhibit the transformation of the ZrO_2_ crystal type and greatly alleviate the decrease in bonding force caused by the volume change in the coatings. The bonding force between the coatings and the matrix is gradually strengthened with the increase in the Y doping amount, and the adhesion gradually improves [44]. However, too-high Y doping will lead to the stabilization effect of the crystalline form being poor, while the increase in the thickness of the coating and the increase in the brittleness of the coating will also make the coating densification poor and decrease the bonding force with the matrix, resulting in cracking or even peeling phenomena. Therefore, when the Y doping amount is 10 wt%, the coating has the best comprehensive physical properties, and the 10YSZ coating has the strongest bonding force with the matrix and a high hardness level.

### 3.5. Antioxidant Behavior of Coatings

**Figure 8:**
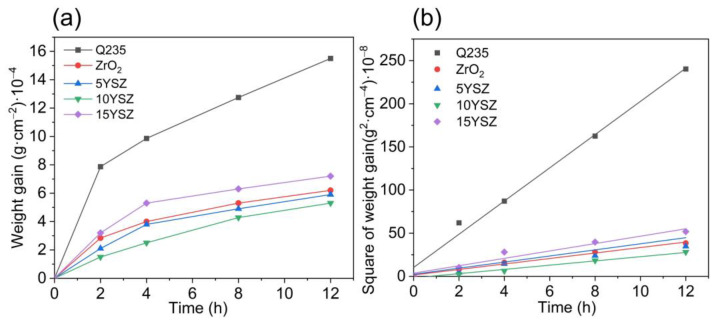
Graph of antioxidant property test results of samples: (**a**) weight gain as a function of time, (**b**) weight gain squared as a function of time.

From Figure 8a, it can be seen that the oxidation kinetic curves of both matrix specimens and coated specimens show parabolic shapes, and Figure 8b shows that the square of the weight gain of the specimens shows a linear relationship with time. The above results indicate that the oxides generated during the heating process can cover the surface densely and will not crack due to excessive internal stress, and the thickening of the oxide film on the surface of the matrix and the coating have a blocking effect on the oxidation, which prevents further oxidation of the matrix. This also indicates that there is a better bonding force between the coating and the matrix [45,46].

With the increase in Y doping in the coatings, the mass of the sample weight gain first decreased and then increased at the same time, and the oxidation rate of the samples first slowed down and then accelerated. This indicates that the antioxidant protection ability of the coating increases and then decreases with the increase in Y doping, and the 10YSZ coating has the best antioxidant performance, while the 15YSZ has the worst antioxidant performance, and even the performance is not as good as that of the ZrO_2_ coating. Combined with the SEM morphology of the coatings and the results of the bonding test, it can be found that the 10YSZ coating has the best densification and bonding; the weight gain of the specimen is mainly attributed to the oxidation of the matrix, and the dense coating can slow down the rate of the oxidation of the matrix at high temperatures, so the dense 10YSZ coating has the best antioxidant performance.

### 3.6. Electrochemical Hydrogen Permeation Performance of Coatings

**Figure 9:**
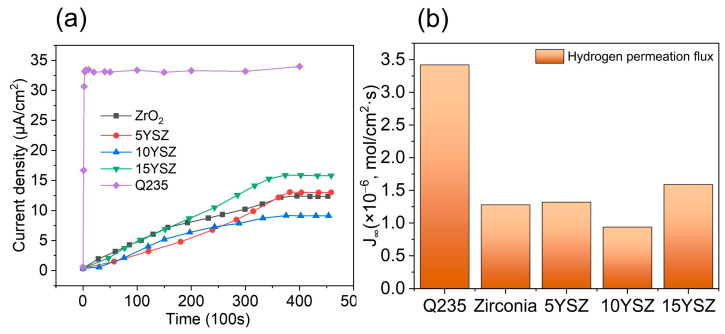
(**a**) Electrochemical hydrogen permeation curves and corresponding (**b**) hydrogen permeation flux curves of the matrix and coatings.

From the electrochemical hydrogen permeation curves in Figure 9a, it can be seen that both the ZrO_2_ coating and the YSZ coating have a good protection effect on the matrix. The steady-state hydrogen permeation current density of the samples protected with the coatings decreased significantly relative to the steady-state current density of 33.0 μA/cm^2^ for the pure matrix. Comparing the electrochemical hydrogen permeation curves of YSZ coatings with different Y doping amounts, it is seen that the hydrogen barrier performances of the coatings are not linearly correlated with the Y doping amount, and the hydrogen barrier performance of the coatings shows an increasing and then decreasing trend as the doping amount increases. The variation in steady-state hydrogen permeation flux for different samples is shown in Figure 9b, and the data related to the hydrogen permeation test are shown in Table 3, which indicate that the coating can effectively reduce the permeation and diffusion of hydrogen into the matrix. The 10YSZ coating had the lowest steady-state hydrogen permeation current density of 9.13 μA/cm^2^, and the steady-state hydrogen permeation current density decreased by 26.1% compared to the ZrO_2_ coating, while the steady-state hydrogen permeation current densities of 5YSZ and 15YSZ were 15.34 μA/cm^2^ and 12.84 μA/cm^2^, respectively. Compared to the matrix, the 5YSZ, 10YSZ, and 15YSZ coatings resulted in a 61.1%, 72.3%, and 53.5% improvement in the hydrogen barrier performance of the specimens, respectively.

From the theory related to Y doping to stabilize the crystal shape, the doping of Y^3+^ produces oxygen vacancies, causing lattice deformation, and the combination of oxygen ion vacancies and Zr^4+^ cations is the main reason for making the tetragonal phase stable at room temperature [40,47]. It has been shown that the stabilization of the tetragonal phase is only controlled by oxygen ion vacancies, and a small concentration of oxygen ion vacancies associated with Zr ions can be effectively generated by doping appropriate amounts of Y ions. However, with the further increase in Y doping, the continuous increase in the oxygen vacancy concentration will lead to increase in the distortion of the coordination layer of ZrO_2_ crystals, and at this time, the local coordination number inside the crystals may appear to be much lower than eight, which is not conducive to the stabilization of the ZrO_2_ crystalline form. From the analysis of the physical properties of the sol, the continuous increase in the viscosity of the sol with the excess increase in Y doping affects the coating quality and thickness of the coating, which ultimately leads to an increase in coating defects and cracks and a decrease in the bonding of the coating to the matrix [48,49]. The hydrogen barrier performance of the coatings decreases as H atoms preferentially pass through the defects of the coatings. There is a high degree of consistency between the changes in antioxidant performance and hydrogen barrier performance of coatings with different Y contents, as both are more sensitive to the densities and integrity of the coatings.

## 4. Conclusions

In this study, composite coatings YSZ with different doping amounts of Y element were prepared by the sol-gel method, and the results through multifaceted characterization and performance evaluation are as follows.

(1) XRD and SEM-EDS analyses show that the rare earth element Y was successfully doped into the ZrO_2_ coating. Y can stabilize the tetragonal phase zirconia existing at high temperatures to a room temperature environment, alleviate the cracking problem of the coating due to the volume change triggered by the crystalline transition, and improve the densification of the coating. Meanwhile, the addition of yttrium can refine the grain size of the oxide; as the Y doping increased from 5 wt% to 15 wt%, the average grain size of the oxides decreased by about 31%, 37%, and 60%, respectively.
(2) Compared with the ZrO_2_ coating, the hardness, adhesion, and antioxidant performances of the YSZ coating show a tendency to increase and then decrease with the increase in Y doping, and the YSZ coating with a Y doping of 10 wt% has the best overall performance. When the Y doping amount was lower than 10 wt%, the uniformity and continuity of the coating surface gradually improved with the increase in yttrium doping, and the hardness was improved, with the hardness level increasing from B to 2H and the adhesion increasing from 4B to 5B level. The surface quality of the coating decreased and cracks and defects increased after the Y doping exceeded 10 wt%. Although the hardness level was further increased to 4H, the coating was more brittle at this time and was prone to peeling off, and the adhesion level decreased to 3B.
(3) After heating at 500 °C for 12 h, the antioxidant performance of the ZrO_2_ coatings was enhanced by 60% compared to the pure substrate, and the antioxidant performance of the 5YSZ, 10YSZ, and 15YSZ coatings was enhanced by 61.9%, 65.8%, and 53.5%, respectively. Meanwhile, the steady-state hydrogen permeation current density of the ZrO_2_ coating was reduced by 62.5% compared to the matrix, and the steady-state hydrogen current density of the 5YSZ, 10YSZ, and 15YSZ coatings was reduced by 61.1%, 72.3%, and 53.5%, respectively.

In conclusion, this study demonstrates that the addition of the appropriate amount of Y element can effectively improve the hydrogen barrier property and other comprehensive properties of ZrO_2_ coating. This provides an experimental basis and theoretical reference for further industrialized application of oxide coatings in many fields in the hydrogen energy industry chain, especially in nuclear energy and aerospace, which require hydrogen permeation protection at high temperatures.

## Figures and Tables

**Table 1:** Chemical composition of Q235 mild steel.

Element	C	Si	Mn	P	S	Fe
Contents (wt%)	0.16	0.18	0.72	0.016	0.018	Bal.

**Table 2:** Heat treatment process parameters for coatings.

Temperature Range (°C)	Heating Rate (°C/min)	Terminal Holding Time (min)
RT–200	1	35
200–400	1	90
400–550	3	120

**Table 3:** Summary of hydrogen permeation results of the matrix, ZrO_2,_ and YSZ coatings.

Sample	Q235	ZrO_2_	5YSZ	10YSZ	15YSZ
I_ss_ (μA/cm^2^)	33.01	12.36	12.84	9.13	15.34
J_∞_ (mol/cm^2^·s)	3.42 × 10^−6^	1.28 × 10^−6^	1.33 × 10^−6^	0.95 × 10^−6^	1.59 × 10^−6^

## Data Availability

The raw data supporting the conclusions of this article will be made available by the authors on request.

## References

[1] Bondarenko V.L., Ilyinskaya D.N., Kazakova A.A., Kozlovtsev P.S., Lavrov N.A., Razenko E.A. (2022). Introduction to Hydrogen Energy. Chem. Pet. Eng..

[2] Han X., Zhang X., Yan H., Kang J., Li J., Zhang H. (2021). Current Situation and Prospect of Global Hydrogen Energy Industry Policy. Electr. Power Inf. Commun. Technol..

[3] Huo X., Wang J., Jiang L., Xu Q. (2016). Review on key technologies and applications of hydrogen energy storage system. Energy Storage Sci. Technol..

[4] Niu M., Xiao Y., Liu F., Zhao P., Zhao B. (2018). Influences of Renewable Energy on Hydrogen Storage System and Its Control Strategy. Electr. Power Constr..

[5] Teng X., Zhang G., Hu C., Zhu C., Yu D., Liu D., Liu S. (2022). Analysis on hydrogen energy economy and low cost of hydrogen source in typical cities of China. Chem. Ind. Eng. Prog..

[6] Sundaram S., Ram G.D.J., Amirthalingam M. (2023). Metallurgical and mechanical properties of hydrogen charged carbide-free bainitic weld metals. Int. J. Hydrogen Energy.

[7] Guan H., Lin Z., Li Y., Liu Q., Xing Y., Wang J., Wang X. (2017). Hydrogen embrittlement susceptibility of the X70 pipeline steel substrate and weld in simulated coal gas containing hydrogen environment. Chin. J. Eng..

[8] Zhao W., Zhang T., Zhao Y., Sun J., Wang Y. (2016). Hydrogen permeation and embrittlement susceptibility of X80 welded joint under high-pressure coal gas environment. Corros. Sci..

[9] Fowler J.D., Chandra D., Elleman T.S., Payne A.W., Verghese K. (1977). Tritium diffusion in Al_2_O_3_ and BeO. J. Am. Ceram. Soc..

[10] Yao Z., Suzuki A., Levchuk D., Chikada T., Tanaka T., Muroga T., Terai T. (2009). Hydrogen permeation through steel coated with erbium oxide by sol-gel method. J. Nucl. Mater..

[11] Heo H.S., Shin D.H., Kim S.J. (2023). Effects of CrN and TiN Coating by Hydrogen Embrittlement of Aluminum Alloys for Hydrogen Valves of Hydrogen Fuel Cell Vehicles on Mechanical Properties. Corros. Sci. Technol. Korea.

[12] Zhou C., He M., Xiao S., Shi K., Wu H., Jiang S., Chen G., Wu C. (2020). Review on hydrogen permeation barrier coatings on stainless steels. Chem. Ind. Eng. Prog..

[13] Wang B., Sun X., Liu E., Liu L., Ma W., Shi Y., Huang P., Luo Y. (2024). Preparation and Hydrogen Barrier Property of Fe_x_Al_y_/Al/Al_2_O_3_ Composite Coating on X80 Steel Surface. Met. Mater. Int..

[14] Huang J., Xie H., Luo L.M., Zan X., Liu D.G., Wu Y.C. (2020). Preparation and properties of FeAl/Al_2_O_3_ composite tritium permeation barrier coating on surface of 316L stainless steel. Surf. Coat. Technol..

[15] Yao Z., Suzuki A., Levchuk D., Terai T. (2007). Sic coating by rf sputtering as tritium permeation barrier for fusion blanket. Fusion Sci. Technol..

[16] Wang P.X., Liu J., Wang Y., Shi B.G. (2000). Investigation of SiC films deposited onto stainless steel and their retarding effects on tritium permeation. Surf. Coat. Technol..

[17] Lakdhar I., Alhussein A., Capelle J., Creus J. (2021). Al-Ti-W alloys deposited by magnetron sputtering: Effective barrier to prevent steel hydrogen embrittlement. Appl. Surf. Sci..

[18] Wang J., Ling Y., Lu Z., Zhang M., Zhou Q., Wang R., Li Y., Zhang Z. (2019). Hydrogen interaction characteristics of a Cr_2_O_3_-Y_2_O_3_ coating formed on stainless steel in an ultra-low oxygen environment. Int. J. Hydrogen Energy.

[19] Kulsartov T.V., Hayashi K., Nakamichi M., Afanasyev S.E., Shestakov V.P., Chikhray Y.V., Kenzhin E.A., Kolbaenkov A.N. (2006). Investigation of hydrogen isotope permeation through F82H steel with and without a ceramic coating of Cr_2_O_3_-SiO_2_ including CrPO_4_ (out-of-pile tests). Fusion Eng. Des..

[20] Han S., Li H., Wang S., Jiang L., Liu X. (2010). Influence of silicon on hot-dip aluminizing process and subsequent oxidation for preparing hydrogen/tritium permeation barrier. Int. J. Hydrogen Energy.

[21] He D., Li S., Liu X., Zhang C., Yu Q., Wang S., Jiang L. (2014). Preparation of Cr_2_O_3_ film by MOCVD as hydrogen permeation barrier. Fusion Eng. Des..

[22] Feng J., Dan M., Jin F., Chen M., Shen L., Tong H., Zhang G. (2016). Preparation and Properties of Alumina Coatings as Tritium Permeation Barrier by Plasma Electrolytic Oxidation. Rare Met. Mater. Eng..

[23] Zhang W., Zhu C., Yang J., Chen Q., Shi K., Wang L., Feng Y., Feng K., Duan Z., Li Y. (2021). Aluminum phosphate sealing to improve deuterium permeation resistance of α-Al_2_O_3_ coating prepared by MOD method. Surf. Coat. Technol..

[24] Wang Z.G., Chen W.D., Yan S.F., Fan X.J., Xu Z.G. (2022). Characterization of ZrO_2_ ceramic coatings on ZrH_1.8_ prepared in different electrolytes by micro-arc oxidation. Rare Met..

[25] Ju H., Chen W., Yan S., Liu T., Liu F., Ma W. (2019). Zirconia Hydrogen Permeation Barrier Fabricated by Sol-Gel Method with Different Solvents. Chin. J. Rare Met..

[26] Siripurapu R.K., Szpunar B., Szpunar J.A. (2014). Molecular Dynamics Study of Hydrogen in α-Zirconium. Int. J. Nucl. Energy.

[27] Barbour O., Crocombette J.P., Schuler T., Tupin M. (2020). Ab-initio calculations of hydrogen diffusion coefficient in monoclinic zirconia. J. Nucl. Mater..

[28] Haurat E., Crocombette J.P., Schuler T., Tupin M. (2022). Hydrogen diffusion coefficient in monoclinic zirconia in presence of oxygen vacancies. Int. J. Hydrogen Energy.

[29] Hatano Y., Zhang K., Hashizume K. (2011). Fabrication of ZrO_2_ coatings on ferritic steel by wet-chemical methods as a tritium permeation barrier. Phys. Scr..

[30] Meetei S.D., Singh S.D., Sudarsan V. (2012). Polyol synthesis and characterizations of cubic ZrO_2_:Eu^3+^ nanocrystals. J. Alloys Compd..

[31] Cong Y., Li B., Yue S., Fan D., Wang X.J. (2009). Effect of Oxygen Vacancy on Phase Transition and Photoluminescence Properties of Nanocrystalline Zirconia Synthesized by the One-Pot Reaction. J. Phys. Chem. C.

[32] Chen Y.X., Liu W.M. (2002). Preparation and tribological properties of sol-gel zirconia thin films stabilized with ceria. Mater. Lett..

[33] Araki W., Arai Y. (2010). Oxygen diffusion in yttria-stabilized zirconia subjected to uniaxial stress. Solid State Ion..

[34] Chen D., Mei D., Li Y., Chen L., Wang H., Huang W., Wang L., Zhu S., Guan S. (2023). Protective nature of cerium-based oxides coating against Mg corrosion in Hanks’ balanced salt solution. Corros. Sci..

[35] Su X., Li X., Cai T., Zhang X. (2017). Study on Preparation Process and Solid Solution Crystal Type of Yttria Stabilized Zirconia by Reverse Coprecipitation Method. China’s Ceram..

[36] Szajna E., Moskal G., Tupaj M., Dresner J., Dudek A., Szymański K., Tomaszewska A., Trzcionka-Szajna A., Mikuśkiewicz M., Łysiak K. (2024). The influence of laser remelting on microstructural changes and hardness level of flame-sprayed NiCrBSi coatings with tungsten carbide addition. Surf. Coat. Technol..

[37] Veldhuis S.A., Brinks P., Ten Elshof J.E. (2015). Rapid densification of sol-gel derived yttria-stabilized zirconia thin films. Thin Solid Film..

[38] Criado J.M., Gonzalez M., Dianez M.J., Perez Maqueda L.A., Malek J. (2007). Influence of environment and grinding on the crystallisation mechanism of ZrO_2_ gel. J. Phys. Chem. Solids.

[39] Mandal S., Kumar C.J.D., Kumar D., Syed K., Van Ende M.A., Jung I.H., Finkeldei S.C., Bowman W.J. (2020). Designing environment-friendly chromium-free Spinel-Periclase-Zirconia refractories for Ruhrstahl Heraeus degasser. J. Am. Ceram. Soc..

[40] Bartuli C., Bertamini L., Matera S., Sturlese S. (1995). Investigation of the formation of an amorphous film at the ZrO_2_-Y_2_O_3_/nicocraly interface of thermal barrier coatings produced by plasma spraying. Mater. Sci. Eng. A.

[41] Wu S., Zhang H. (2015). Preparation and Structural Analysis of Y_2_O_3_ Stabilized ZrO_2_ Coating Material. Rare Met. Mater. Eng..

[42] Huang W., Yang J., Meng X., Cheng Y., Wang C., Zou B., Khan Z., Wang Z., Cao X. (2011). Effect of the organic additions on crystal growth behavior of ZrO_2_ nanocrystals prepared via sol-gel process. Chem. Eng. J..

[43] Chen K., Song P., Hua C., Zhou Y., Huang T., Li C., Lu J. (2018). Effect of YSZ-dopant on microstructure and hardness property of the Al_2_O_3_-40%TiO_2_ plasma sprayed coating. Mater. Res. Express.

[44] Tiwari S.K., Adhikary J., Singh T.B., Singh R. (2009). Preparation and characterization of sol-gel derived yttria doped zirconia coatings on AISI 316L. Thin Solid Film..

[45] Yonggui W., Erni Q.I.U., Minghui D. (2007). Characterization Analysis of Oxidation Kinetics of Aluminized Coating on Ni-base Superalloy. Hot Work. Technol..

[46] Li Z.Y., Zhang J.Y., Bao R., Hu H.J., Fei B.J. (2009). Experimental study on adaptivity of oxidation kinetics equations in APS thermal barrier coatings. J. Aerosp. Power.

[47] Hebert V., His C., Guille J., Vilminot S., Wen T.L. (1991). Preparation and characterization of precursors of Y_2_O_3_ stabilized ZrO_2_ by metal-organic compounds. J. Mater. Sci..

[48] Soo M.T., Kawamura G., Muto H., Matsuda A., Lockman Z., Cheong K.Y. (2013). Design of hierarchically meso-macroporous tetragonal ZrO_2_ thin films with tunable thickness by spin-coating via sol-gel template route. Microporous Mesoporous Mater..

[49] Zima T.M., Baklanova N.I., Belyaeva E.I., Lyakhov N.Z. (2006). Preparation of ZrO_2_ and ZrO_2_-Y_2_O_3_ coatings on silicon carbide fibers. Inorg. Mater..

